# Supplementary material for: Longer metaphase and fewer chromosome segregation errors in modern human than Neanderthal brain development
Source: Sci Adv. 2022 Jul 29;8(30):eabn7702. doi: 10.1126/sciadv.abn7702 (PMC9337762; doi:10.1126/sciadv.abn7702)
Supplement: Supplementary file 1 — Figs. S1 to S8 Tables S1 and S2 [file sciadv.abn7702_sm.pdf]

Science Advances
AAAS

Supplementary Materials for

Longer metaphase and fewer chromosome segregation errors in modern human than Neanderthal brain development

Felipe Mora-Bermúdez *et al.*

Corresponding author: Felipe Mora-Bermúdez, mora@mpi-cbg.de; huttner@mpi-cbg.de;
Svante Pääbo, paabo@eva.mpg.de

Sci. Adv. **8**, eabn7702 (2022)
DOI: 10.1126/sciadv.abn7702

This PDF file includes:

Figs. S1 to S8
Tables S1 and S2

Supplementary figures and figure legends

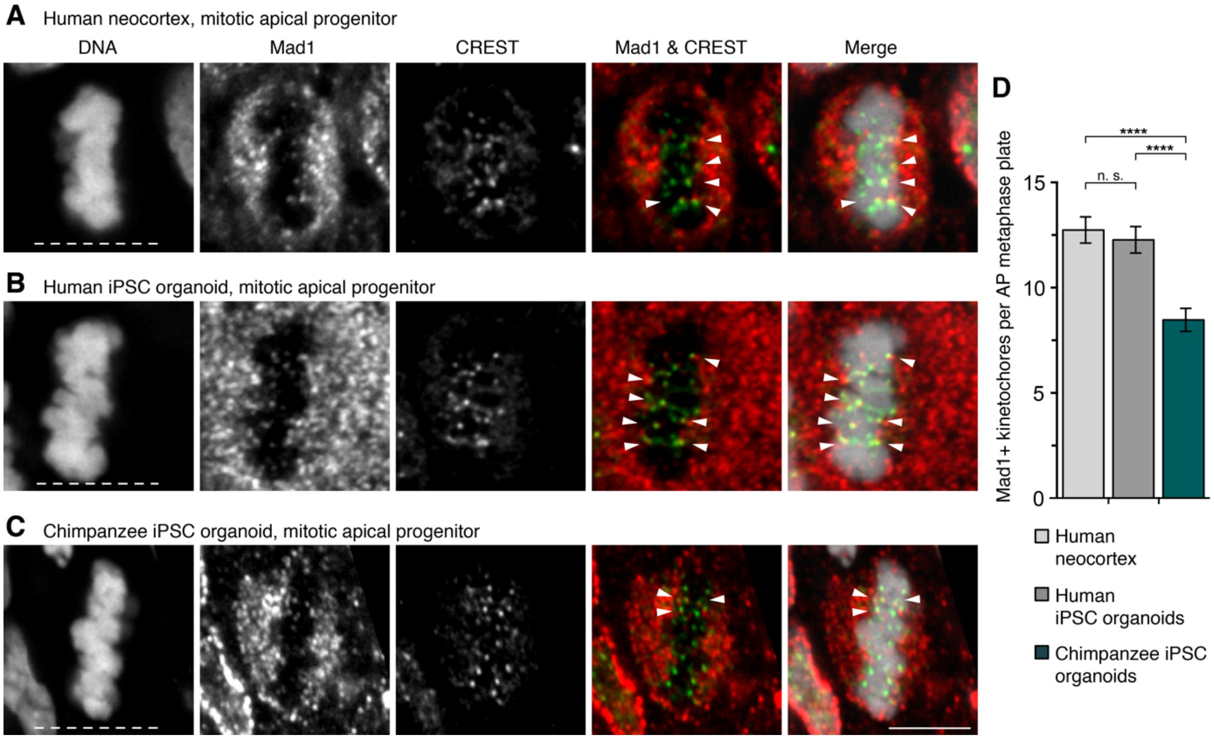

Supplementary Figure 1. More Mad1-positive kinetochores in modern human than chimpanzee APs. (**A–C**) Mitotic APs in metaphase stained with DAPI and immunostained for the SAC marker Mad1 (red in merges) and the kinetochore marker CREST (green in merges). Arrowheads, overlap of Mad1 and CREST immunoreactivity within the metaphase plate. (**A**) GW11 modern human neocortex; (**B**) day 30 modern human iPSC-derived cerebral organoid; (**C**) day 31 chimpanzee iPSC-derived cerebral organoid. White dashed lines, ventricular surface. Scale bar, 5 µm. (**D**) Quantification of Mad1-positive kinetochores per AP metaphase plate for the tissues in A–C (GW11-12, days 30-32). Data are the mean ± SEM of ≥ 37 APs from three independent experiments with three neocortex samples, and ≥ 50 APs from ≥ 3 independent experiments with ≥5 organoids per species. Brackets with ****, $p < 0.0001$; n. s., non-significant (Kruskal-Wallis test with Dunn's multiple comparisons correction).

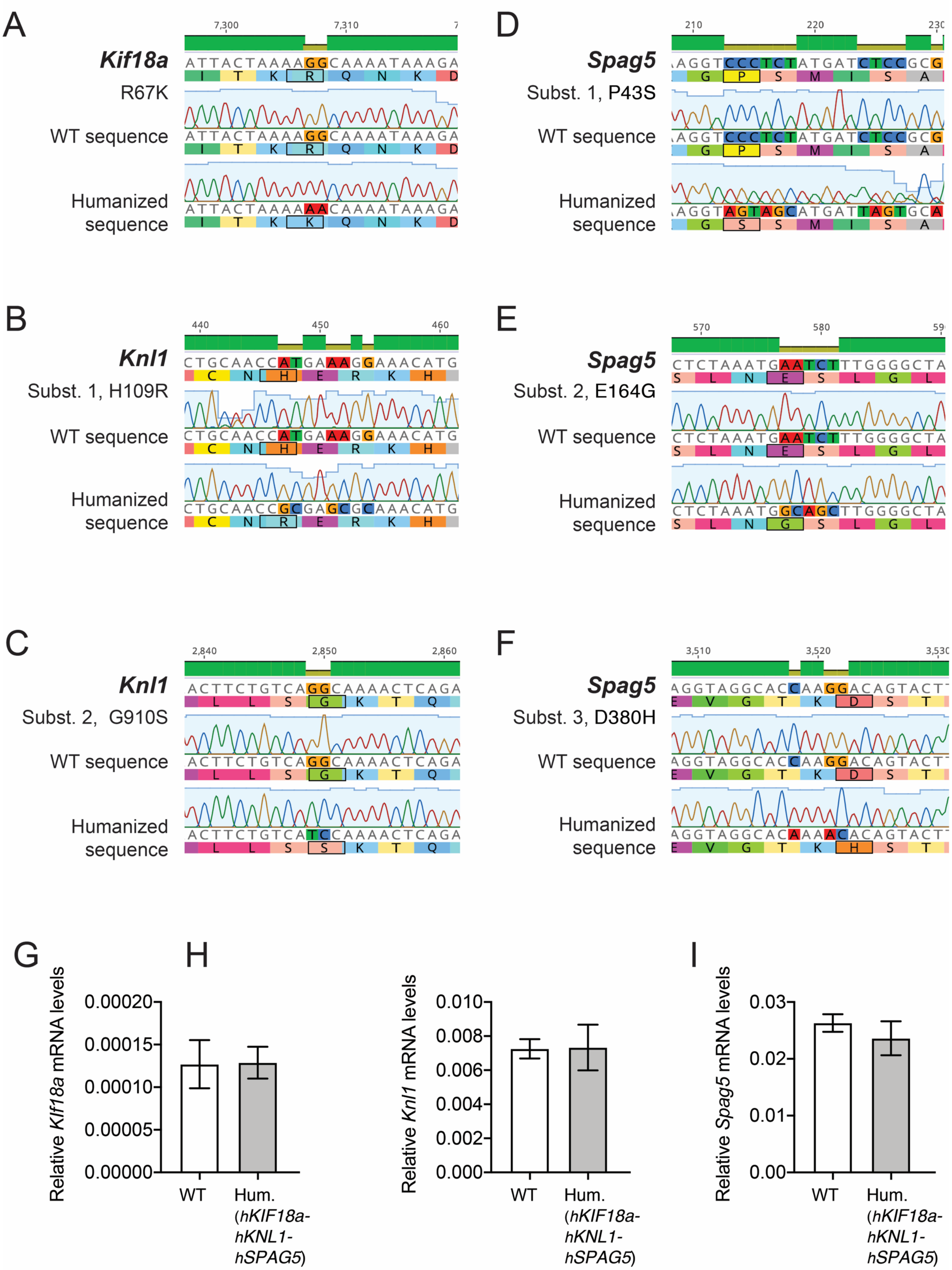
A
Kif18a
R67K
WT sequence
Humanized sequence
D
Spag5
Subst. 1, P43S
WT sequence
Humanized sequence
B
Knl1
Subst. 1, H109R
WT sequence
Humanized sequence
E
Spag5
Subst. 2, E164G
WT sequence
Humanized sequence
C
Knl1
Subst. 2, G910S
WT sequence
Humanized sequence
F
Spag5
Subst. 3, D380H
WT sequence
Humanized sequence
G
H
I
Relative Kif18a mRNA levels
0.00020
0.00015
0.00010
0.00005
0.00000
Relative Knl1 mRNA levels
0.010
0.008
0.006
0.004
0.002
0.000
Relative Spag5 mRNA levels
0.03
0.02
0.01
0.00
WT
Hum. (hKIF18a-hKNL1-hSPAG5)

Supplementary Figure 2. Similar mRNA expression levels of *Kif18a*, *Knl1* and *Spag5* in wt and humanized E11.5 mouse telencephalon. (**A**) *Kif18a* sequences in wt and humanized (h or Hum, hKif18a-hKnl1-hSpag5 (see also Figure 3A) E11.5 mouse telencephalon, aligned with the mouse *Kif18a* gene. (**B**, **C**) *Knl1* sequences for the amino acid (aa) substitution positions 1 and 2 (Subst.1 & 2) in WT and humanized 11.5 mouse telencephalon, aligned with the mouse *Knl1* gene. (**D-F**) *Spag5* sequences for the aa substitution positions 1, 2 and 3 (Subst. 1, Subst. 2 & Subst. 3) in WT and humanized E11.5 mouse telencephalon, aligned with the mouse *Spag5* gene. (**G-I**) qPCR analysis of the expression of *Kif18a* (G), *Knl1* (H) and *Spag5* (I), relative to the expression of *Actb,* in the WT and humanized E11.5 mouse telencephalon. Data are the mean of four biological replicates, each of which contains one or two telencephala. Error bars indicate SD, $p = 0.9210$ (G), $p = 0.9207$ (H), $p = 0.1603$ (I) (two-tailed unpaired Student's t-tests).

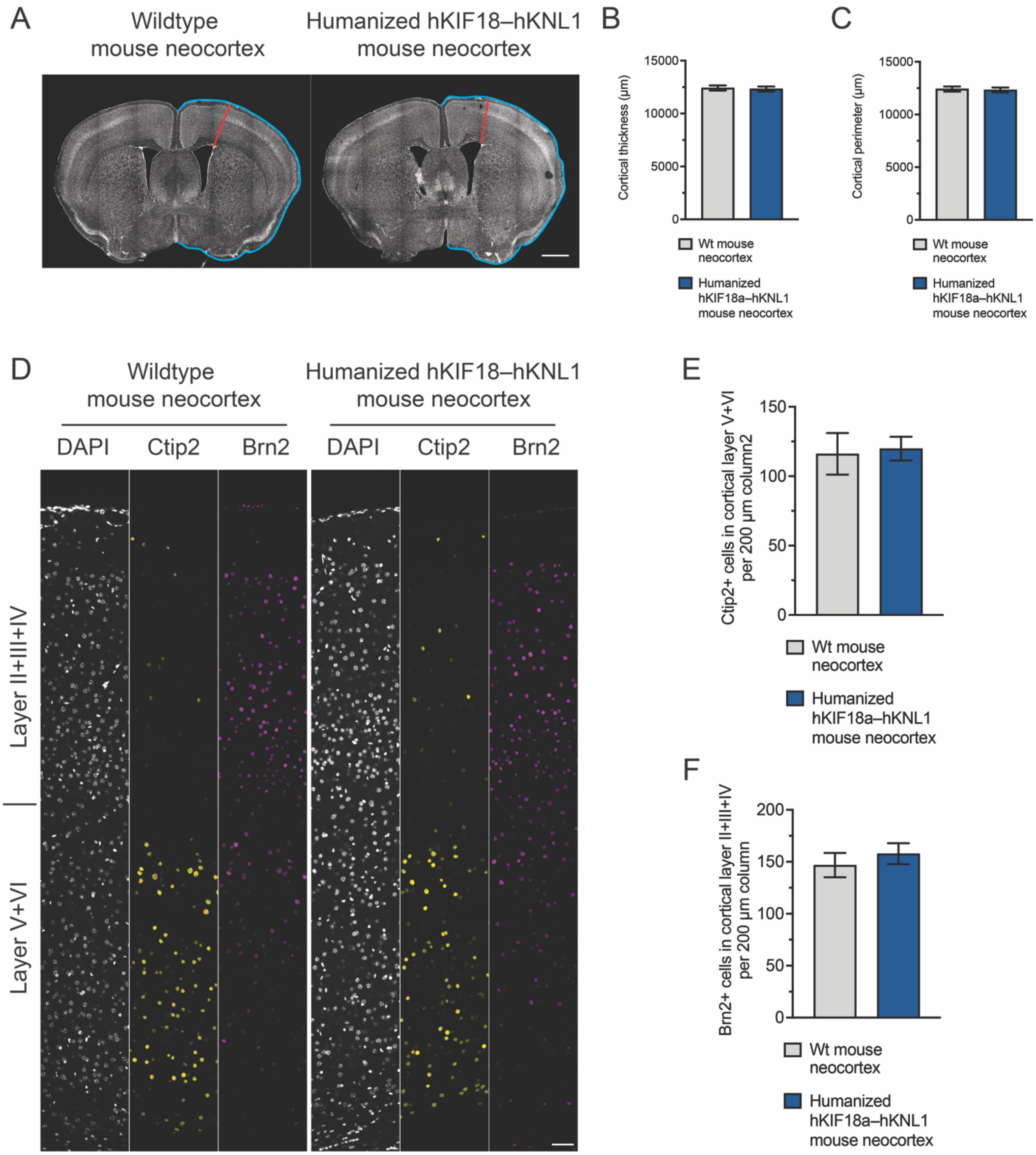

Supplementary Figure 3. No differences in cortical thickness and cortical perimeter, nor in deep-layer and upper-layer neuron numbers in adult mice humanized for KIF18a and KNL1 compared to wildtype mice. (**A**) Representative DAPI staining (white) of 8 week-old adult neocortex of wildtype mice and mice humanized for KIF18a and KNL1 illustrating the

measurements of cortical thickness (red lines) and perimeter (blue lines). Quantification of cortical thickness (**B**) and perimeter (**C**). (**D**). Representative immunofluorescence for Ctip2 (yellow) and Brn2 (magenta), combined with DAPI staining (white), of 8 week-old adult neocortex of wildtype mice and mice humanized for KIF18a and KNL1. (**E, F**). Quantification of Ctip2+ (**E**) and Brn2+ (**F**) and neurons in a 200 µm-wide field of 8 week-old adult neocortex. Images are single optical sections (**A, D**). Scale bar, 1 mm (**A**), 20 µm (**D**). Data are the mean of 6 wildtype mice (3 male and 3 female) and 6 mice humanized for KIF18a and KNL1 (3 male and 3 female). Error bars indicate SD, $p > 0.05$ (two-tailed unpaired Student's *t*-test, **B, C, E, F**).

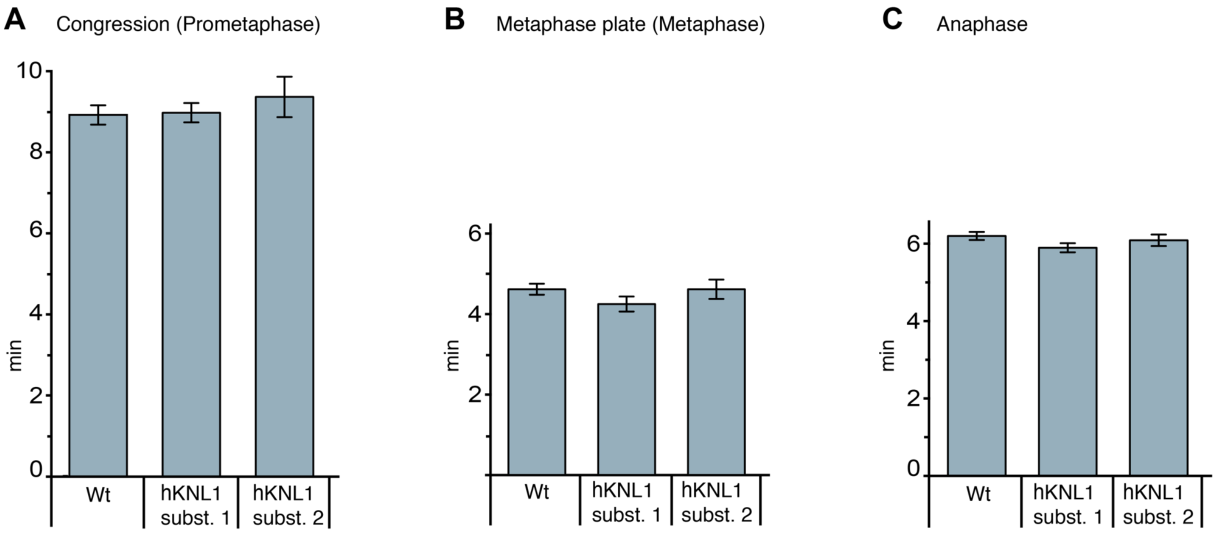

Supplementary Figure 4. Neither of the two individual *KNL1* amino acid substitutions alters metaphase duration. Times (**A**) between the start of chromosome congression and metaphase plate onset (referred to as "Congression" or "Prometaphase"); (**B**) between metaphase plate onset and chromatid segregation onset (referred to as "Metaphase plate" or "Metaphase"); and (**C**) between the onset of chromatid segregation and onset of chromosome decondensation (referred to as "Anaphase"). Data is for APs in the neocortical tissues of two mouse lines gene-edited using CRISPR/Cas9 (see Materials and Methods) to substitute, in each line, one of the two wt (Neandertal-like) amino acid residues of KNL1, shown in Figure 3A, for the respective modern human variant. The data for wt mice from Figure 3 is included for comparison. Data are the mean ± SEM of ≥ 53 APs from 3 independent experiments, with a total of ≥ 4 neocortices, for each of the six lines; $p > 0.05$ for all comparisons (Kruskal-Wallis test with Dunn's multiple comparisons correction).

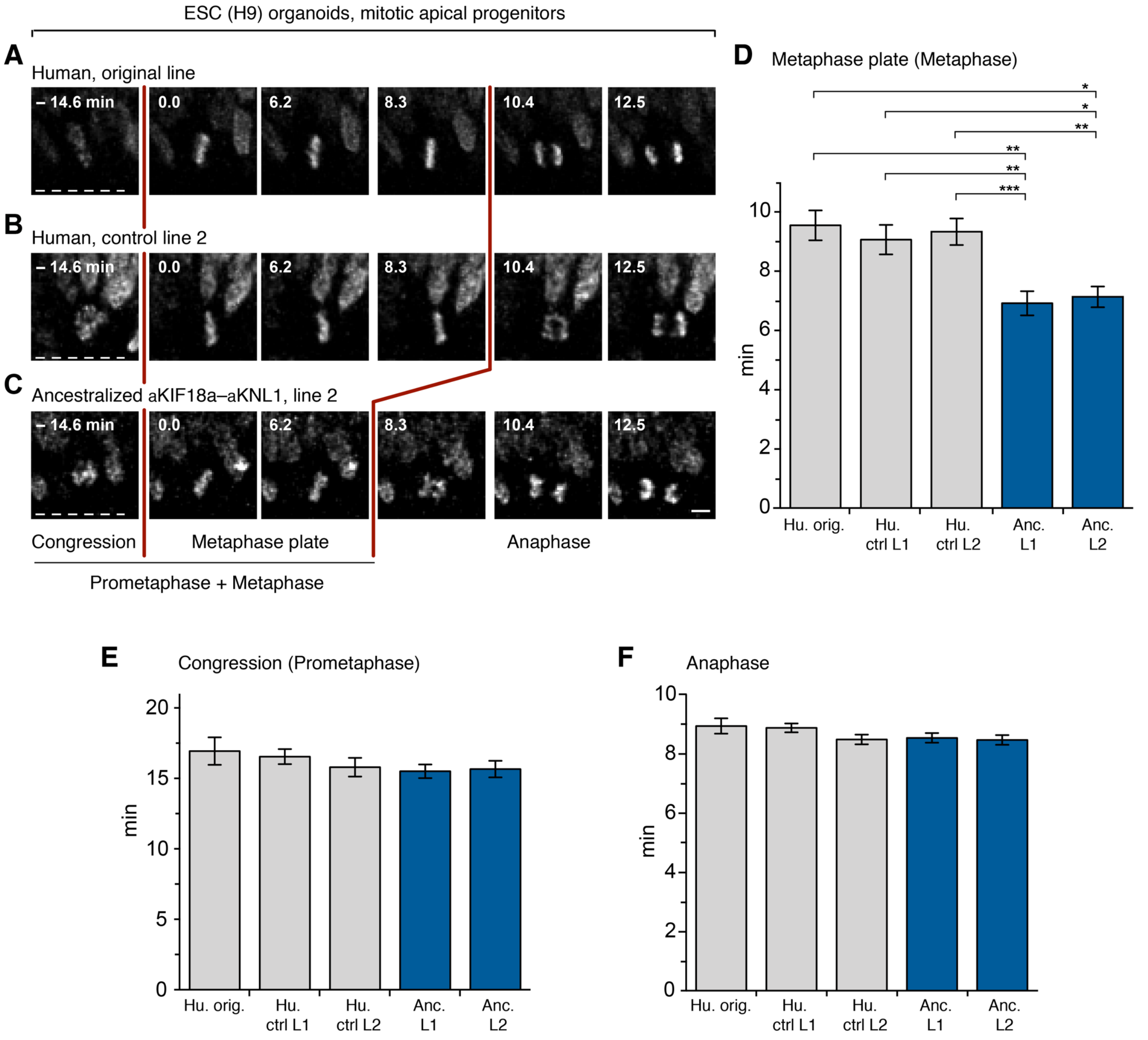

Supplementary Figure 5. AP metaphase shortening in organoids ancestralized for *KIF18a* and *KNL1* is independent of the individual cell line used. (**A-C**) Live-tissue imaging of the indicated mitotic phases of apical progenitors in organotypic slice cultures of day 27-30 cerebral organoids grown from the indicated ESC lines. (**A**) original, non-edited modern human H9 line (Hu. orig. in D-F); (**B**) the control line 2 (line 1 is shown in Figure 4B); (**C**) the ancestralized aKIF18a–aKNL1 line 2 (line 1 is shown in Figure 4C). Zero (0) min is metaphase plate onset. Time-lapse intervals are 2.08 min. Red lines indicate the duration of metaphase. White dashed lines, ventricular surface. Scale bar, 5 µm. Times (**D**) between metaphase plate onset and

chromatid segregation onset (referred to as “Metaphase plate” or "Metaphase"); (**E**) between chromosome congression onset and metaphase plate onset (referred to as “Congression” or “Prometaphase”); and (**F**) between chromatid segregation onset and chromosome decondensation onset (referred to as “Anaphase”), for apical progenitors in organoids grown from each of the indicated cell lines (crtl, control; L, line, Anc., ancestralized). Data are the mean ± SEM of ≥ 52 APs from ≥ 2 independent experiments, with a total of ≥4 organoids, for each of the five lines. Brackets with *, $p < 0.05$; ** $p < 0.01$; ***, $p < 0.001$ (Kruskal-Wallis test with Dunn’s multiple comparisons correction).

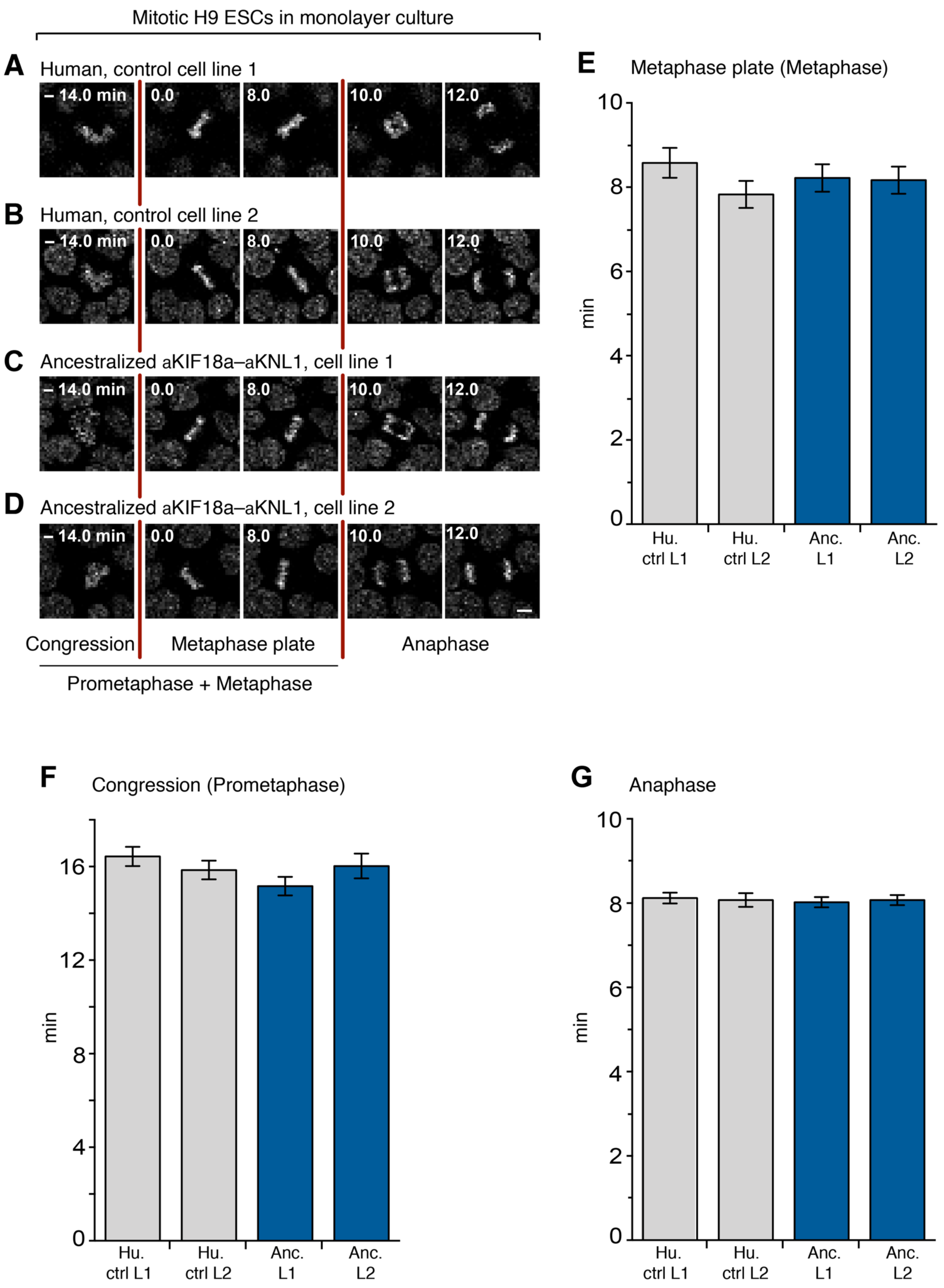

Supplementary Figure 6. ESCs ancestralized for *KIF18a* and *KNL1* do not show metaphase shortening in monolayer culture. (**A-D**) Live-tissue imaging of the indicated mitotic phases of the indicated H9 ESCs in monolayer culture. (**A**) Control ESC line 1; (**B**) control ESC line 2; (**C**) ancestralized aKIF18a–aKNL1 ESC line 1; (**D**) ancestralized aKIF18a–aKNL1 ESC line 2. Zero

(0) min is metaphase plate onset, time-lapse intervals are 2 min. Red lines indicate the duration of metaphase. Scale bar, 5 µm. Times (**E**) between metaphase plate onset and chromatid segregation onset (referred to as "Metaphase plate" or “Metaphase”); (**F**) between chromosome congression onset and metaphase plate onset (referred to as “Congression” or “Prometaphase”); and (**G**) between chromatid segregation onset and chromosome decondensation onset (referred to as “Anaphase”), for each of the four H9 ESC lines described in (A–D; Hu., human; Anc., ancestralized; ctrl, control, L, line). Data are the mean ± SEM of ≥ 81 cells from three independent experiments, for each of the four lines.

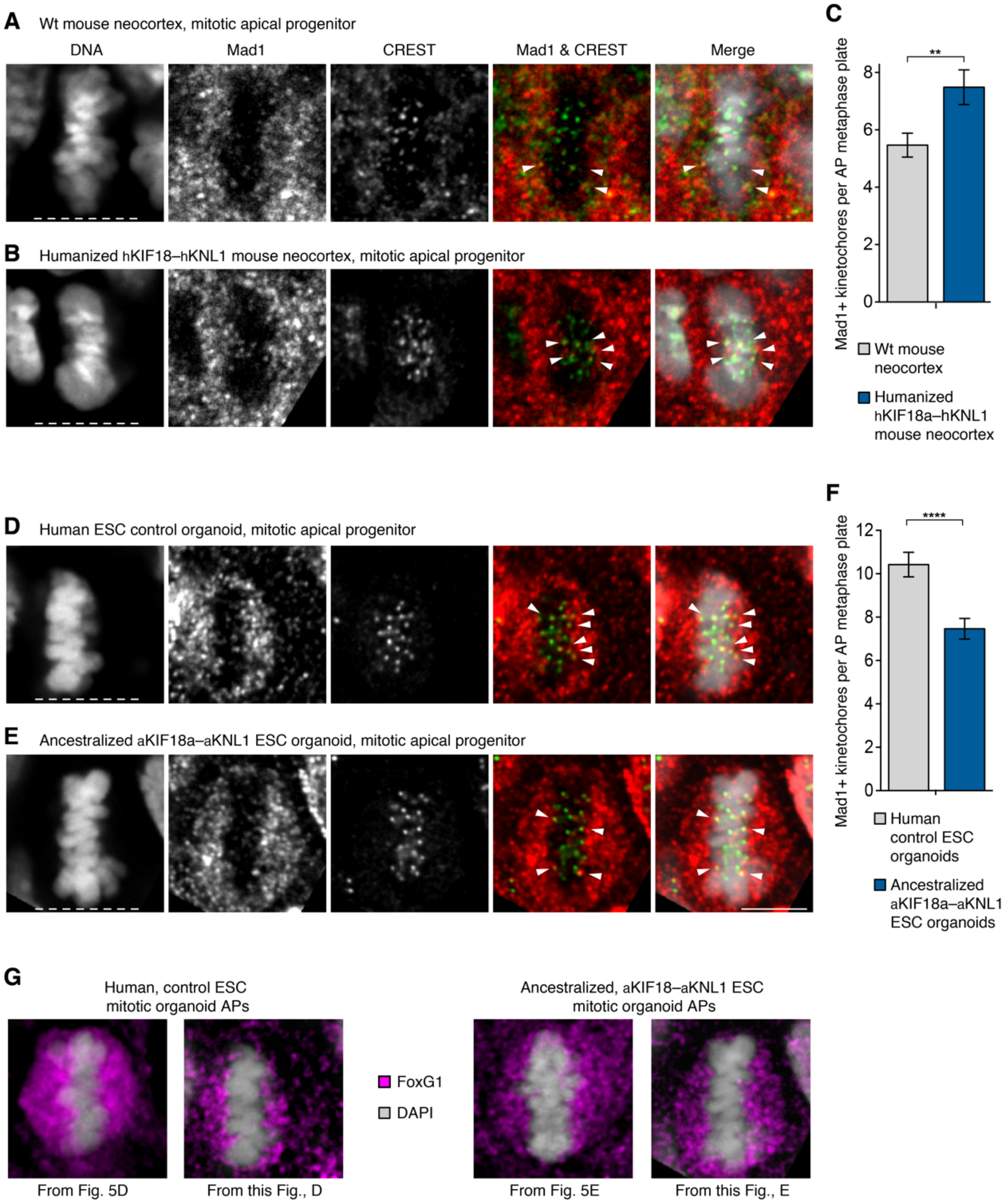

Supplementary Figure 7. More Mad1-positive kinetochores in mice humanized for *Kif18a* and *Knl1*, and fewer in organoids ancestralized for *KIF18a* and *KNL1*. (**A, B, D, E**) Mitotic APs in metaphase stained with DAPI and immunostained for the SAC marker Mad1 (red in merges) and the kinetochore marker CREST (green in merges). Arrowheads indicate overlap of BubR1 and CREST immunoreactivity within the metaphase plate. (**A**) Neocortex of E11.5 wt

(ancestral-like) mouse; (**B**) neocortex of E11.5 mouse humanized for *Kif18a* and *Knl1*; (**D**) modern human non-edited day 28 cerebral organoid (control line 2); (**E**) day 29 organoid ancestralized for KIF18a and KNL1 (edited line 2). White dashed lines, ventricular surface. Scale bar, 5 µm. (**C**) Number of Mad1-positive kinetochores per AP metaphase plate in mouse neocortex as described in (A, B). Data are the mean ± SEM of ≥ 32 APs from three independent experiments, with a total of ≥ 5 neocortices, for each of the two types of mice. Bracket with **, p <0.01 (Mann-Whitney U test). (**F**) Number of Mad1-positive kinetochores per AP metaphase plate for day 27-30 organoids (e.g., D, E). Data are the mean ± SEM of ≥ 65 APs from five independent experiments, with a total of ≥ 7 organoids, for each of the two ESC lines. Bracket with ****, p < 0.0001 (Mann-Whitney U test). (**G**) Mitotic APs (immuno)stained for FoxG1 (magenta) and DAPI (grey) in the indicated types of organoids. From left to right: from Figure 5D, this figure panel D, from Figure 5E, and this Figure panel E.

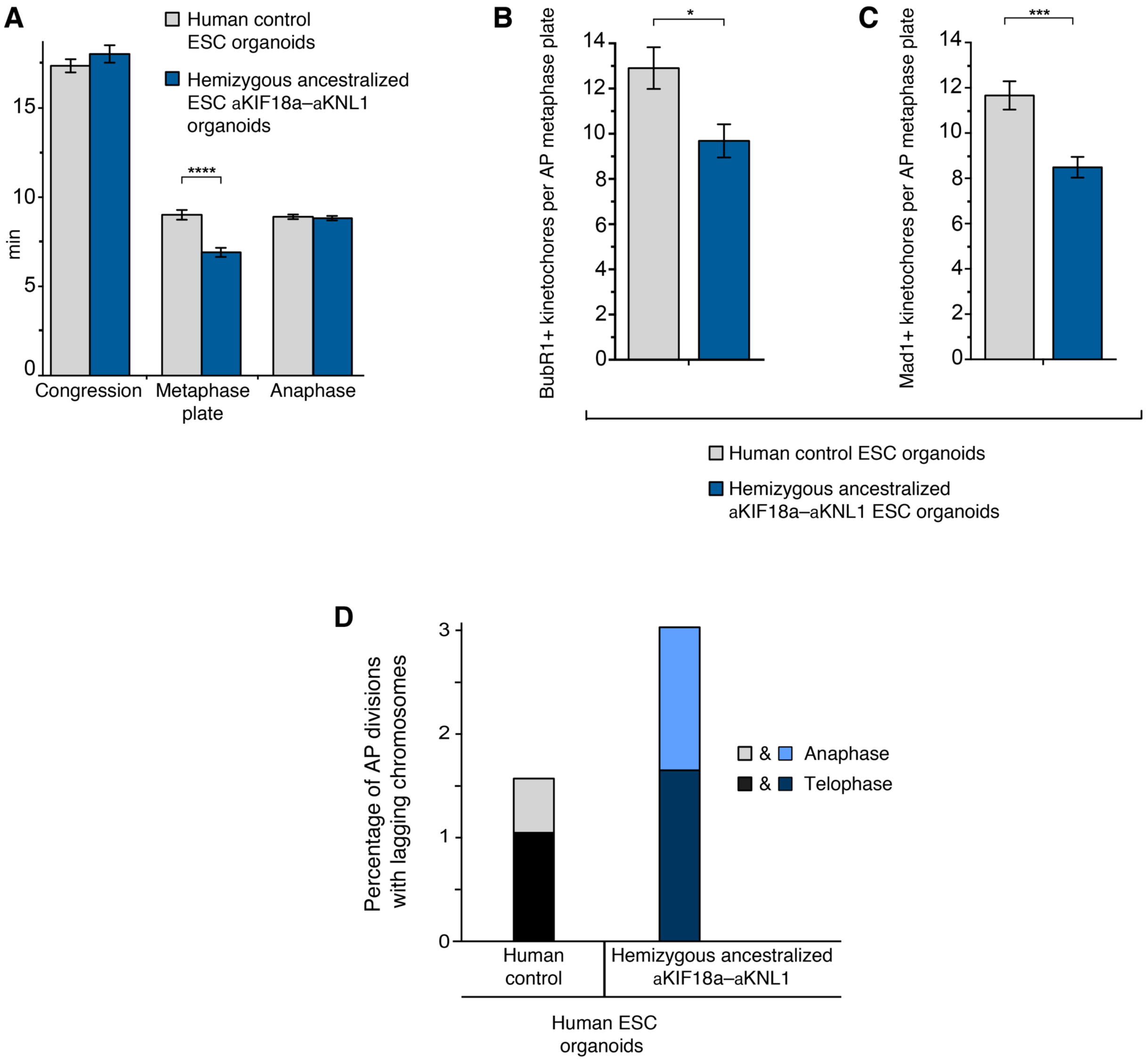

Supplementary Figure 8. Organoids from ESC lines hemizygous for the ancestralized *KIF18a* or *KNL1* show results similar to those from homozygous lines. All panels show data similar to the panels indicated below, but with cerebral organoids from two H9 ESC lines, one where the gene *KIF18a* is hemizygous and edited to the ancestral, Neandertal-like state and the gene *KNL1* is homozygously edited, and the other where the gene *KNL1* is hemizygous and edited to the ancestral, Neandertal-like state and the gene *KIF18a* is homozygously edited. The results obtained with these two ESC lines were pooled. (**A**) Similar to Figure 4D. Times of

“Congression”, “Metaphase plate” and “Anaphase” for APs in the two types of organoids. Data are the mean ± SEM of ≥134 APs from four independent experiments, with ≥7 organoids, for each of the lines. Bracket with ****, $p < 0.0001$ (Mann-Whitney U test). (**B**) Similar to Figure 5F. Number of BubR1-positive kinetochores per AP metaphase plate in the two types of organoids. Data are the mean ± SEM of ≥35 APs from four independent experiments, with a total of ≥ 7 organoids, for each of the two lines. Bracket with *, $p < 0.05$ (Mann-Whitney U test). (**C**) Similar to Supplementary Figure 7F. Number of Mad1-positive kinetochores per AP metaphase plate in the two types of organoids. Data are the mean ± SEM of ≥ 53 APs from five independent experiments, with a total of ≥ 7 organoids, for each of the two lines. Bracket with ***, $p < 0.001$ (Mann-Whitney U test). (**D**) Similar to Figure 6I right. Percentages of AP divisions with lagging chromosomes in the two types of organoids. Data are the sum of ≥ 362 AP divisions from six experiments for each of the two types of organoids. The percentages for telophase (dark shade) and anaphase (light shade) are indicated separately.

Supplementary tables

Target	Primer	Primer sequence	Fragment length (bp)	Temperature (°C) d-a-e	Cycling (sec) d-a-e	Cycle sec	purpose
Kif18A, aa1, exon 2.	kif18A-seq-fwd	GAAGACTTATGTCACCGCATGA	456	95/55/72	30/30/30	35	PCR + seq
	kif18A-seq-rev	TCTGCTGAAATCTCAAAACTGC					
	kif18A-fwd-wt	CTACAAATTTGATATTACTAAAA**GG**	296	94/57/72	30/30/30	35	allele-specific GT
	kif18A-fwd-mut	CTACAAATTTGATATTACTAAAA**AAC**					
	kif18A-rev	TCTGCTGAAATCTCAAAACTGC					
Knl1, aa1, exon 8.	casc5-sg1-seq-fwd	TAGGCACACATGCAGGTAGAAC	691	98/62/72	15/20/20	30	PCR + seq
	casc5-sg1-seq-rev	TCAACTCCATACACTCATTGCC					
	casc5-sg1-fwd	ATTTGGAGCTAGAGAATTGGC	295	94/60/72	30/30/30	35	allele-specific GT
	casc5-sg1-rev-wt	ATTTGCATGTTT**CCTTTCATGG**					
	casc5-sg1-rev-mut	TCATTTGCATGTTT**GCGCTCGC**					
Knl1, aa2, exon 8.	casc5-sg2-seq-fwd	TGGATATCACCAAGAGTTGCAC	672	98/62/72	15/20/30	30	PCR + seq
	casc5-sg2-seq-rev	CAAAACTGAAGCCCTTTCTGTC					
	casc5-sg2-fwd-wt	CTGAATGAACTTCTGTCA**GGC**	351	94/60/72	30/30/30	35	allele-specific GT
	casc5-sg2-fwd-mut	CTGAATGAACTTCTGTCA**TCC**					
	casc5-sg2-rev	TCTGGTGACATCCAGATCAC					
Spag5, aa1, exon 2.	spag5-sg1-seq-fwd	CTCACACTCGCTTTCTCTCTCA	500	98/56/72	15/20/140	28	PCR + seq
	spag5-sg1-seq-rev	CAGGAAAATTCTAACTACCCCA					
	spag5-sg1-fwd-wt	TCCCTCTATGATCTCCGCG	wt: 369 mut: 383	94/60/72	30/30/30	35	

	spag5-sg1-fwd-mut	GATTCAGGGAAAGGTAGTAGC					allele-specific GT
	spag5-sg1-rev	GGATATGGTTTCAACTCTTGG					
Spag5, aa2+3, exon 3.	spag5-sg2+3-seq-fwd	GATCCAACTCCTGAAAGCAACT	1100	98/64/72	15/20/140	30	PCR
	spag5-sg2+3-seq-rev	CTTGTAGCTGGTGGGAAAGAAC					
	spag5-sg2-seq	GCTGAAGGAGAAAGACGAG		56			internal seq
	spag5-sg3-seq	ACTTGGATGTCCCCATTGGC		56			Internal seq
Spag5, aa2, exon 3.	spag5-sg2-fwd	AAGATGCAACAACTCATCTCC	wt: 331 mut: 329	94/60/72	30/30/30	35	allele-specific GT
	spag5-sg2-rev-wt	ATCTTCTAGCCCCAA**AGATTC**					
	spag5-sg2-rev-mut	TCTTCTAGCCCCAA**GCTGC**					
Spag5, aa3, exon 3.	spag5-sg3-fwd-wt	TGGAGGTAGGCAC**CAAGG**	wt: 310 mut: 312	94/60/72	30/30/30	35	allele-specific GT
	spag5-sg3-fwd-mut	ACTGGAGGTAGGCAC**AAAAC**					
	spag5-sg3-rev	TGTGTACTACTGTCTCGAGC					

Supplementary table 1. Overview of primers and sequencing conditions for genotyping of gene-edited mice. Primers used (a) for the sequencing of CRISPR/Cas9 events (PCR+seq); and (b) for allele-specific genotyping (GT) in wt and humanized mice. Aa amino acid. Initial denaturation: 2 min at 95°C, d-a-e: denaturation – annealing – extension during PCR cycling, extension: 5 min at 72°C.

	Reverse transcriptase intact			*Reverse transcriptase heat-inactivated*		
**Template**	***KIF18a***	***KNL***	***PPIB***	***KIF18a***	***KNL***	***PPIB***
RNA - ancestralized line 1	28.5	27.8	26.8	N/A	N/A	N/A
RNA - ancestralized line 2	27.7	28.4	27.2	N/A	N/A	N/A
RNA - non-edited line 1	27.1	27.9	26.7	N/A	N/A	N/A
RNA - non-edited line 2	28.6	29.0	27.9	N/A	N/A	N/A
H9-KR line	27.5	28.1	27.1	N/A	N/A	N/A
RNA extraction blank	N/A	N/A	N/A	N/A	N/A	N/A
DNA HeLa cells	N/A	N/A	N/A	N/A	N/A	N/A
water blank	N/A	N/A	N/A	N/A	N/A	N/A

Supplementary Table 2. Similar *KIF18a*, *KNL* and *PPIB* mRNA levels in ancestralized and control cell lines. Numerical values correspond to cycle threshold in RT-qPCR; N/A indicates that there was no amplification of the targeted region.
